# Model for Early Detection of Breast Cancer in Low-Resource Areas: The Experience in Peru

**DOI:** 10.1200/JGO.17.00006

**Published:** 2018-01-26

**Authors:** Carolyn Bain, Tara Hayes Constant, Ines Contreras, Ana Maria Burga Vega, Jose Jeronimo, Vivien Tsu

**Affiliations:** **Carolyn Bain**, **Tara Hayes Constant**, **Ines Contreras**, **Jose Jeronimo**, and **Vivien Tsu**, PATH, Seattle, WA; and **Ana Maria Burga Vega**, Instituto Regional de Enfermedades Neoplasicas - Norte, Trujillo, Peru.

## Abstract

**Purpose:**

Late-stage breast cancer detection should be something of the past; however, it is still all too common in low-resource areas, including Peru, where 57% of women diagnosed with cancer are diagnosed at stage III or IV disease. Early detection of breast cancer is feasible in low-resource semirural and rural areas where mammography is rarely accessible.

**Methods:**

PATH collaborated with Peruvian health institutions at local, regional, and national levels to design and implement a model of care for the early detection of breast cancer in Peru. The model includes training health promoters for community outreach, professional midwives in clinical breast exam, doctors to perform fine-needle aspiration biopsy sampling with ultrasound to triage, and patient navigators to ensure patients follow through with treatment.

**Results:**

In a northern region of Peru, 400 individuals, including health promoters, midwives, doctors, and volunteers, received early-detection training in two phases. In Peru, local health professionals continue to refine and improve methods and materials using locally available resources, and the Peruvian health information system now includes specific breast cancer detection categories. Despite challenges and limited resources, the model is effective, and partnership with government health administrations improves health systems and benefits the population.

**Conclusion:**

Given the absence of screening mammography, the public health challenge is to bring breast cancer early detection and diagnostic services closer to women’s homes and to ensure appropriate follow-up and care. The model is eminently transferable with appropriate adaptation and should now be tested in other settings within and outside of Peru.

## INTRODUCTION

Rates of breast cancer are increasing in low- and middle-income countries (LMICs) partly as a result of an epidemiologic shift caused by lifestyle changes, later reproductive age, and longer life expectancy.^1^ This is the case in Latin America, where breast cancer incidence is on the rise.^2^ At the same time, there is a clear disparity in breast cancer mortality, particularly in lower-resource areas outside of metropolitan centers, because women are more often diagnosed in later stages of breast cancer when treatment is less likely to be successful.^3^ In Peru, 57% of women diagnosed with cancer are diagnosed with stage III and IV disease at the national level^4,5^; these women require more intensive and expensive treatment and have significantly poorer outcomes.

Fortunately, the Peruvian government recognized this serious problem and launched a national program in 2012 that targeted five cancers, including breast cancer, to address cancer prevention, diagnosis, and treatment.^6^ The program provides these services free of charge to the poorest Peruvians. Cancer treatment is offered by the national cancer hospital, two regional cancer institutes, and major regional hospitals. Leveraging the Peruvian government’s commitment to cancer care, PATH, a nonprofit global health organization, collaborated with local and national health institutions, including the Ministry of Health, the National Cancer Institute of Peru, and the Northern Regional Cancer Institute, to establish a model of care for the early detection of breast cancer in low-resource communities. The WHO defines early detection as having two elements: early diagnosis of people with symptoms and screening of people without apparent symptoms.^7^ This work describes the process of implementing this model, which is designed to ensure that low-income women in rural areas can access treatment that is now both locally available and financially accessible in Peru. This model of care targets areas of Peru with minimal access to mammography. The cost for a mammogram is prohibitive for most rural and semirural women, and the few available machines are largely located in metropolitan centers. Furthermore, whereas Peru nationally has 55 mammogram machines in public hospitals, only four machines are located in urban areas of the northern region. There are 305,229 women older than age 50 years who live in this region.^8^ Mammography need far exceeds supply.^9^ In a setting with such poor access to mammography, clinical breast exams (CBEs) that are integrated into the primary health care infrastructure provide a resource-appropriate screening modality.^10^

In conjunction with colleagues from the Breast Health Global Initiative (BHGI), and using the BHGI resource-based guidelines as a planning tool, PATH worked with Peruvian partners to develop and pilot targeted screening interventions where they could have the greatest effect. Duggan et al^11^ describe the theory behind the BHGI resource-based service stratification and use the model developed in Peru to elucidate these concepts.

During its initial phases, the PATH-organized pilot model for early detection focused on a small-town health network in the northern region—an area similar to many communities in Peru.

## METHODS

**Fig 1:**
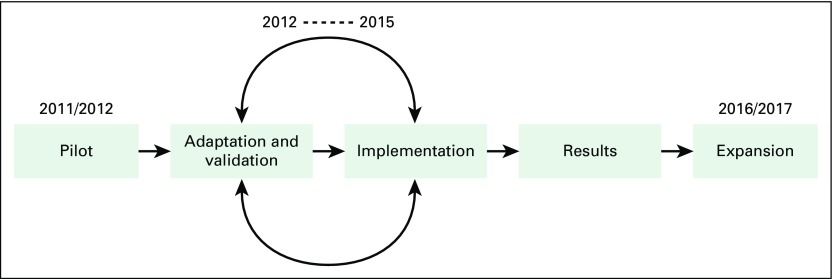
Timeline for the development of an early detection model

Over a 6-year period, PATH worked with international experts, Peruvian cancer specialists, and regional partners to develop, pilot, adapt, and validate the comprehensive and resource-appropriate model of care for the early detection of breast cancer in low-resource settings and specifically tailored it to Peru (Fig 1).

### Design and Pilot

The model now has five components (Table 1): health education by community health promoters to raise awareness among women about early breast cancer symptoms and the need for breast cancer screening, even without symptoms; CBE by professional midwives in a primary care setting; ultrasound triage for palpable masses detected with CBE; fine-needle aspiration (FNA) biopsy sampling by general physicians and gynecologists at local community hospitals, with referral if positive; and patient navigation to assure that women with a referral to tertiary care receive timely attention.

**Table 1:**
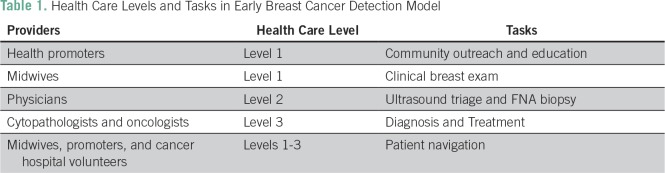
Health Care Levels and Tasks in Early Breast Cancer Detection Model

CBE is a cornerstone of the model of care, but must be complemented with adequate follow-up and the appropriate use of limited specialty health care services. The innovative approach of having general physicians carry out FNA sampling in this setting enables the government to decentralize services and detect breast cancer at earlier stages in more rural and remote areas. FNA is performed at the primary care level, and the specimen is interpreted by a trained cytopathologist at a cancer hospital. It is a model that is suitable for LMICs that can provide adequate diagnostic and cancer treatment services for women who have been identified as being in need of them.

PATH coordinated international teams of stakeholders to develop and validate educational and communication materials for health promoters to use in the community, as well as training curricula and manuals for CBE, ultrasound triage, FNA sampling, and patient navigation trainings. All health services that were offered as part of the care model were delivered by regular government employees paid by the local public health system. The project adapted and finalized checklists and clarified roles and responsibilities for supervision of the program in breast health. A process for changing the national health information system (HIS) to incorporate key breast indicators was also initiated as part of the project. Peru’s HIS remains only partly electronic, with information generated at the lower levels of the health care system still collected manually. PATH project staff worked closely with the Ministry of Health’s statistics teams to pilot modifications to the national HIS that would allow for the tracking of critical indicators in the national HIS.

###  Adaptation and Validation of the Model of Care

**Fig 2:**
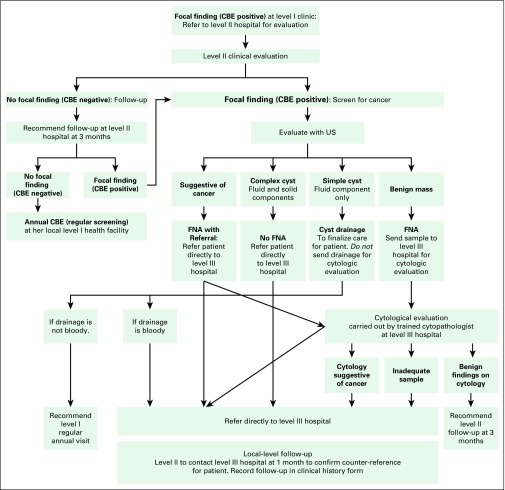
Flowchart for ultrasound (US) triage and fine-needle aspiration (FNA) for women with positive clinical breast exam (CBE).

Over the course of 6 years, notable refinements were incorporated into the care model to address challenges related to the limited availability of specialty services, loss to follow-up, and age-appropriate use of screening exams. One refinement was the introduction of ultrasound triage of palpable masses to decrease the number of FNA biopsies required, thereby conserving limited cytopathology resources and reducing unnecessary procedures for women (Fig 2). An algorithm was developed to guide trained gynecologists and general physicians such that women with masses suggestive of cancer that were found on ultrasound triage were sent directly to the regional cancer institute for additional management and final diagnosis. Benign masses were sampled for confirmation, and benign cysts drained with return for follow-up in 1 year (Fig 2).

Another refinement was the training of volunteers in patient navigation. Early in the pilot implementation, midwives noted that women often failed to start treatment once they received a diagnosis. At least two of 10 women who were diagnosed with breast cancer delayed treatment and required intensive follow-up to re-engage them. Common reasons for this delay were fear about the illness and potential abandonment by their spouse. To address this issue, a group of health professionals working in cancer prevention created a patient navigator program to address delays in treatment. This working group included women hospital volunteers, psychologists, social workers, insurance representatives, and former patients with cancer. The training curriculum for patient navigators included the following topics: empowering patients with cancer, effective communication, medical information for breast cancer, health and insurance systems, directory of resources, lifestyle changes, and post-treatment issues.

One particularly appropriate local modification was made to the FNA biopsy–staining process. The head of cytopathology at the national cancer institute recognized that it was more feasible to use hematoxylin instead of toluidine blue to stain samples at regional hospitals when doctors assessed specimen adequacy. Hematoxylin is readily available in the country, whereas toluidine blue must be specially imported into Peru.

Finally, during pilot implementation, the team found that many women who received CBE were younger than age 40 years because breast exams were considered a usual part of reproductive health and prenatal visits. PATH initiated discussions of the issue at the local, regional, and national levels with Peruvian health administration to emphasize the importance of targeting women age 40 to 65 years to decrease the likelihood of detecting benign changes in the breast that occur more frequently in younger women, conserve the time of physicians and midwives, and minimize strain on the newly implemented referral system for FNA and diagnostic procedures. The entire set of educational and training materials for this care model has been reviewed by the Peruvian School for Breast Health for validation and future national implementation.

## RESULTS

### Pilot

**Fig 3:**
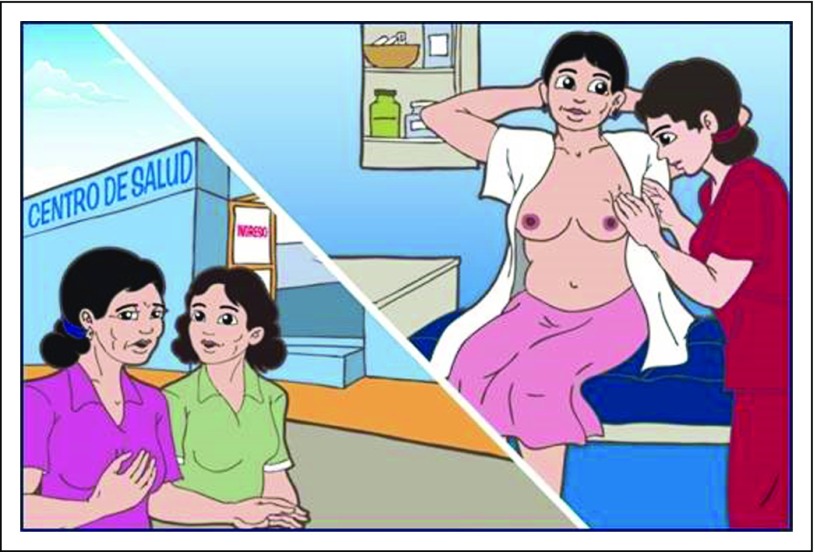
Example of graphic illustrations used in community educational and training materials.

In 2011 and 2012, the initial phase of the pilot took place in one health network. Once the training curriculum and manuals had been developed and approved by the expert advising team, community health promoters and health professionals participated in relevant trainings (Fig 3). Trainers included international experts and Peruvian cancer institute professionals. In total, 48 health promoters, 36 midwives, 19 doctors, and 11 supervisors were trained during this phase of the pilot.

During the pilot implementation period (2011 to 2016), 15,000 women were reached with more than 900 educational sessions. Among the 13,500 women who received CBE, 321 breast abnormalities were found, 114 FNA biopsies were performed, and 10 cancers were identified. Women with cancer then initiated treatment at the regional cancer hospital. After the addition of ultrasound, the need for FNA biopsy was reduced by 65% in the first few months.

As a result of PATH’s analysis of the Peru HIS codes related to breast information, the Ministry of Health incorporated several code changes to capture key variables: first annual CBE, repeat CBE exam, FNA biopsy, and referral. The Ministry of Health has stated that it is considering adapting the existing HIS formats for cancer follow-up, which could unify cancer registry data nationally.

Another important outcome is the implementation of the patient navigation program led by volunteers who successfully steward women through the fragmented and sometimes bewildering process of cancer diagnosis and treatment. Trained navigators empower women to complete treatments and follow-up visits, assist in finding resources, and provide emotional support to women and their families throughout the process. This model has now been replicated in Lima by national cancer institute volunteers. After adapting the training curriculum, volunteers have presented this experience at several conferences and shared lessons that were learned with neighboring Latin American countries (E. Cisneros, personal communication, November 2016).

Perhaps one of the most significant outcomes from the pilot implementation phase was the development of the School for Breast Health in 2013 at Peru’s national cancer institute, in collaboration with PATH, as a center of excellence to focus explicitly on training promoters and health professionals in early breast cancer detection. The School for Breast Health provides national leadership in disseminating training throughout the country and has vetted the model of care that was piloted in the north. It has taken on a supervisory role using the checklists and tools that were developed for implementation. There have already been two examples of the self-seeding of the care model in new geographic areas. In one instance, one of the doctors who was originally trained in the pilot was transferred to a new health network where he continues to apply his knowledge and skills, receiving referrals of women with suspicious breast masses. During a 1.5-year period, this doctor evaluated 75 women, and performed 21 FNA biopsies . In the second case, doctors from the regional cancer institute organized and conducted training on CBE and FNA sampling for six doctors from three nearby health networks. Key elements of the model have been institutionalized at the national level. The Ministry of Health has approved FNA for use in secondary and tertiary health facilities for the early detection and diagnosis of breast cancer.

Frequent turnover of national and regional health administrations and professionals, however, is a common occurrence and can disrupt the continuity of the implementation of the early detection model.

## DISCUSSION

The expansion started in July 2016 and extended services to nine additional health networks in Peru’s northern region. Professionals and community volunteers from 58 primary and secondary level health facilities received training in the five model components for a total of 95 health promoters, 194 professional midwives, eight doctors, and 29 patient navigators trained. Together with PATH, Peruvian experts have continued to refine the procedures, curricula, and manuals as they participate in regional-level trainings.

The local administration of the Ministry of Health in the northern region is an active partner in the expansion phase of the program. It has committed its staff to assist in the coordination of trainings and purchased the ultrasound equipment needed to successfully practice the triage algorithm. The main cancer coordinator for the nine networks uses part of her time to support the implementation of the model by troubleshooting potential roadblocks and managing institutional logistics. There is momentum and motivation to scale the model up in regional networks; midwives, doctors, and health promoters are energized to participate in this model that allows for the early detection of breast cancer for women in their communities. This expansion at the regional level will demonstrate the utility of the care model for the rest of the country. Currently, Peru’s Ministry of Health administrators are eager to see outcomes and subsequently explore implementation options in other regions.

The model for the early detection of breast cancer described here has proven to be effective and is feasible to implement—it is an example of real-life implementation in a low-resource area. With local experts and administrators involved at every step of the process, there is a sense of ownership and a desire to see it sustained and used in more remote rural areas.

Nonetheless, implementation of any new activity faces multiple challenges. Frequent turnover of health administration officials in LMICs creates a difficult environment for the maintenance of stability and sustainability. These changes can result from routine shifts that come with newly elected political parties or power struggles. Peru is no exception to this rule. In the 6 years since the pilot began, there have been five different regional health directors. The head of the national cancer institute has changed twice, and the current Peruvian Minister of Health is the fourth minister to lead the Ministry since the project began. With each of these shifts in power, the priorities and assignment of staff or funding can change. Given this scenario, it is important for international organizations to support capacity building and long-term collaboration.

The lack of databases and unified registries is another serious challenge for innovative programs. In Latin America, there is an overall need to improve cancer surveillance, as well as quality, coverage, and data use.^2^ Better systems for data collection on the impact of these interventions are urgently needed for appropriate evaluation and adaptation. For example, in the pilot phase, it was difficult to track the number of women who came for CBEs as a result of exposure to health promotion sessions.

In addition, because the HIS is not set up for longitudinal tracking of individual women, it is difficult to assess whether women completed referrals. As noted above, changes to HIS that might aid in the evaluation of programs are complex and slow to be adopted—another key area in need of international support and one that would yield long-term benefits.

There are high levels of motivation and engagement among Peruvian health professionals to implement this comprehensive early breast cancer detection model. Having a dedicated coordinator for the logistical and administrative aspects of an early breast cancer detection program is crucial for sustainability. Fortunately, Peru has strong national champions for cancer.

Despite some challenges, this model is sound and there is local, regional, and national engagement in sustaining and continuing to expand the implementation of the model throughout the country. Similar to breast cancer detection programs that have been carried out in Malaysia and Egypt using CBE, this program model resulted in breast cancer downstaging and is feasible and affordable. Programs in Nepal and Malawi that have incorporated CBE have also proven to be effective.^12^

In Honduras, recent opportunistic breast cancer education and screening resulted in increased uptake of services and may result in the earlier detection of breast cancer.^13^

Given the absence of screening mammography, the public health challenge is to bring breast cancer early detection and diagnostic services closer to women’s homes and to ensure appropriate follow-up and care. The model described here is eminently transferable with appropriate adaptation to specific country conditions, and should now be tested in other settings, within and outside of Peru.
